# Challenges and opportunities of the COVID-19 pandemic for perinatal mental health care: a mixed-methods study of mental health care staff

**DOI:** 10.1007/s00737-021-01108-5

**Published:** 2021-04-08

**Authors:** C. A. Wilson, C. Dalton-Locke, S. Johnson, A. Simpson, S. Oram, L. M. Howard

**Affiliations:** 1 Section of Women’s Mental Health, Institute of Psychiatry, Psychology and Neuroscience, King’s College London, London, UK; 2 South London and Maudsley NHS Foundation Trust, London, UK; 3 NIHR Mental Health Policy Research Unit, Division of Psychiatry, University College London, London, UK; 4 Camden and Islington NHS Foundation Trust, London, UK; 5 Health Service and Population Research, Institute of Psychiatry, Psychology and Neuroscience, King’s College London, London, UK; 6 NIHR Mental Health Policy Research Unit, Institute of Psychiatry, Psychology and Neuroscience, King’s College London, London, UK

**Keywords:** Perinatal, Mental health, COVID-19, Coronavirus, Pandemic

## Abstract

The aim of this study was to explore staff perceptions of the impact of the COVID-19 pandemic on mental health service delivery and outcomes for women who were pregnant or in the first year after birth (‘perinatal’ women). Secondary analysis was undertaken of an online mixed-methods survey open to all mental health care staff in the UK involving 363 staff working with women in the perinatal period. Staff perceived the mental health of perinatal women to be particularly vulnerable to the impact of stressors associated with the pandemic such as social isolation (rated by 79.3% as relevant or extremely relevant; 288/363) and domestic violence and abuse (53.3%; 192/360). As a result of changes to mental health and other health and social care services, staff reported feeling less able to assess women, particularly their relationship with their baby (43.3%; 90/208), and to mobilise safeguarding procedures (29.4%; 62/211). While 42% of staff reported that some women engaged poorly with virtual appointments, they also found flexible remote consulting to be beneficial for some women and helped time management due to reductions in travel time. Delivery of perinatal care needs to be tailored to women’s needs; virtual appointments are perceived not to be appropriate for assessments but may be helpful for some women in subsequent interactions. Safeguarding and other risk assessment procedures must remain robust in spite of modifications made to service delivery during pandemics.

## Introduction

Since the advent of the global COVID-19 pandemic in March 2020, numerous concerns regarding its impact on public mental health have been raised. These include the impact of lockdown and social distancing measures, bereavement and a range of other socio-economic stressors (Adhanom Ghebreyesus 2020). Surveys of adults from around the world have reported increased symptoms of anxiety and depression, with many finding women to be at an increased risk (González-Sanguino et al. 2020; Hyland et al. 2020; Pierce et al. 2020). There are also concerns about the impact of the pandemic on those with existing mental illness (Sheridan Rains et al. 2020). Those with severe mental illness are at increased risk of COVID-19 infection due to higher rates of comorbidities such as physical ill health and socio-economic adversities. They are also more vulnerable to the indirect effects of the pandemic such as reduced service provision and social isolation, which may precipitate relapse (Moreno et al. 2020).

Perinatal mental health services in the UK support women during pregnancy and up to 1 year postpartum with a range of mental illnesses, including schizophrenia and bipolar disorder, and the more common mental disorders of anxiety and depression. Pre-pandemic, mental illness affected at least one in five women in the perinatal period and is associated with adverse maternal and child outcomes (Ayers and Shakespeare 2015; Stein et al. 2014). Several factors associated with the pandemic may disproportionately affect this perinatal population already vulnerable to mental ill health. During the early phase of the pandemic, women in the UK were advised to follow stricter social distancing than the general population (UK Government 2020), leading to reduced contact with friends, family and health and social care services (Caparros-Gonzalez and Alderdice 2020). Another factor associated with mental ill health which has increased during the pandemic is violence, abuse and exploitation (Usher et al. 2020). The perinatal period is a time of increased risk for domestic violence and abuse (DVA) and it is associated with adverse maternal and child outcomes (Oram et al. 2017).

There remains limited understanding of the impact of the pandemic on care provided for those with mental illness, including in the perinatal period (The Lancet Psychiatry 2020). However, there are reports of early discharges from inpatient psychiatric units and reduced community service provision, including fewer face-to-face meetings (Moreno et al. 2020). The aim of this study was to explore, among those working in mental health services in the UK, the impact of the pandemic on the mental health care provided to women in the perinatal period.

## Materials and methods

From 22 April 2020 to 12 May 2020, the NIHR Mental Health Policy Research Unit (MHPRU) surveyed staff working across services that provide mental health care in the UK, including the National Health Service (NHS), private services, social care and third sector or voluntary services. The development of the online mixed-methods questionnaire has been previously described and was done in close collaboration with service users and clinicians (Johnson et al. 2020). Both structured and open-ended questions explored staff’s perceptions of problems faced by service users and carers and challenges to care provision. These core questions were followed by additional questions specific to staff working in particular settings and specialities. Recruitment was via social media, for example Twitter, and relevant professional bodies, with a particular emphasis on recruiting staff from diverse groups. The King’s College London Research Ethics Committee approved the study (MRA-19/20-18372).

The sample comprised those in the survey who reported working with pregnant or postnatal women in generic or perinatal specialist services (community teams or inpatient Mother and Baby Units: MBUs) and who completed at least one question from each of the three main sections of questions open to all respondents described above, as per the approach taken by a previous analysis of the full sample of respondents (all mental health staff) (Johnson et al. 2020).

Quantitative data were analysed using Stata 15 (StataCorp 2007) and are presented as descriptive statistics. Qualitative data from open-ended questions were analysed using inductive semantic thematic analysis (Braun and Clarke 2006). Initial coding of data led to the identification of potential themes, which were checked against coded extracts in a recursive process. For the purposes of providing context for the qualitative analysis, several of the authors are practising psychiatrists, some of whom work in perinatal psychiatry, although none was practising in perinatal services at the time of the pandemic. Peer debriefing was employed between author C.A.W., who conducted the qualitative analysis, and author L.M.H. (Lincoln and Guba 1985).

## Results

### Participant characteristics

Of 2180 respondents who completed at least one question from each of the three main sections open to all respondents (3712 started the survey and 1793 reached the end of it), 363 reported working with perinatal service users in generic or specialist services. This sample of 363 was used for the analysis. Characteristics of the sample are presented in Table 1. The majority (330/363; 91.2%) worked in the NHS and in England (299/363; 82.4%). Fifty-four of the staff reported working in a hospital inpatient service, 18 of these in MBUs. 41.6% (151/363) identified as a manager or lead clinician in their service.

**Table 1:** Characteristics of staff working with perinatal women *N* = 363 staff

Characteristic	*n* (%)
Gender	
Female	236 (65)
Male	41 (11.3)
Missing, N/A or prefer not to say	86 (23.7)
Ethnicity	
White	224 (61.7)
Asian	19 (5.2)
Mixed or multiple ethnic groups	10 (2.8)
Black	8 (2.2)
Other	1 (0.3)
Missing, N/A or prefer not to say	101 (27.8)
Profession	
Nurse	117 (32.2)
Psychiatrist	47 (13)
Clinical or counselling psychologist	37 (10.2)
Social worker	22 (6.1)
Occupational therapist	19 (5.2)
Peer support worker	9 (2.5)
Manager	9 (2.5)
Other qualified therapists	57 (15.7)
Other work	45 (12.4)
Missing	1 (0.3)
Sector*	
NHS	330
Voluntary	16
Social care or other local government	14
Private	11
Community or user-led organisations	5
Setting*	
Community mental health team (CMHT)	255
Crisis service	78
Inpatient service	54
Community groups	26
Residential service	5
Crisis house	3
Other	43

^*^Staff could report working in multiple settings or sectors

### Difficulties experienced by users of perinatal mental health services

Staff were asked to rate the relevance, on a five-point scale, of pre-specified problems among service users and carers with whom they were currently in contact. Table 2 displays proportions of staff rating each item as ‘very relevant’ or ‘extremely relevant’; items are ordered by highest proportion first.

**Table 2:** Difficulties rated as very or extremely relevant for mental health service users and their carers identified by mental health staff

	n/N*	%
Lack of access to usual support networks of family and friends	288/363	79.3
Loneliness due to or made worse by social distancing, self-isolation and/or shielding	275/363	75.8
Lack of usual work and activities	245/358	68.4
Worries about getting COVID-19 infection	240/363	66.1
Lack of access to usual support from other services (primary care, social care, voluntary sector)	232/363	63.9
Worries about family getting COVID-19 infection	227/363	62.5
Lack of access to usual support from NHS mental health services	196/362	54.1
Increased risk from abusive domestic relationships	192/360	53.3
Increased difficulties for families/carers	190/360	52.8
Relapse and deterioration in mental health triggered by COVID-19 stresses	182/363	50.1
Increase in reliance on family/family tensions	164/358	45.8
High personal risk of severe consequences of COVID-19 infection (e.g. due to physical health comorbidities)	155/359	43.2
Difficulty engaging with remote appointments by phone or via digital platforms	153/363	42.1
Having to stay at home in poor circumstances or not having a home to go to	142/362	39.2
Difficulty getting food, money or other basic resources	138/362	38.1
Diminished access to physical health care for problems other than COVID-19	131/362	36.2
Difficulty understanding or following current government requirements on social distancing, self-isolation and/or shielding	117/362	32.3
Effects of COVID-19-related trauma	113/360	31.4
Risk of increased drug and alcohol use or gambling	108/360	30.0
Lack of access to or of equitable provision of physical health care for COVID-19	81/362	22.4
Lack of access to medication and to processes for administering and monitoring it	67/360	18.6
Loss of liberty and rights due to changes in implementation of mental health legislation	61/360	16.9
Problems with police or other authorities because of lack of understanding of/ability to stick to current government requirements	42/362	11.6

^*^Total respondents for this item

**Fig. 1:**
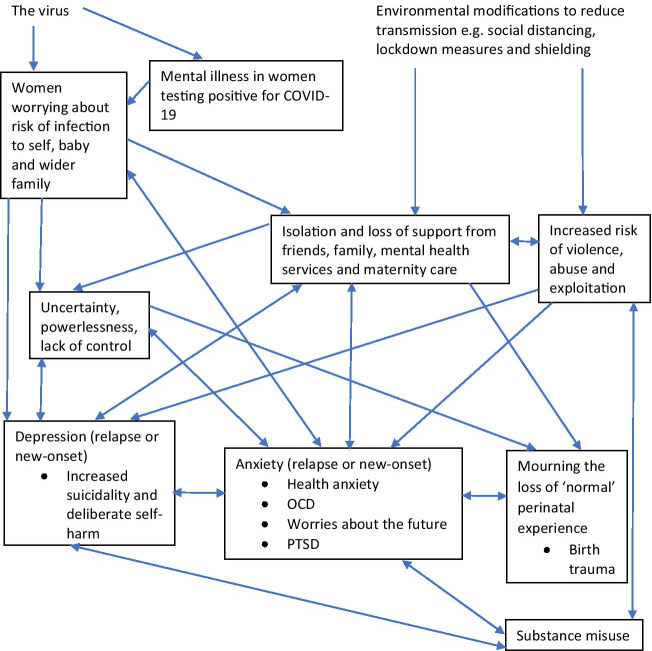
Concerns from staff about areas of difficulty for perinatal service users and their perceived impacts (themes in boxes)

Responses to free text questions revealed similar concerns. Staff were asked for which service users they were especially concerned and new mental health problems arising directly from the pandemic. The majority of staff reported being concerned about women in the perinatal period. Concerns and their impacts grouped as themes and how they inter-relate are displayed in Fig. 1. Difficulties related either to the virus itself or environmental adaptations to reduce transmission. A few staff described cases of mental illness in women testing positive for COVID-19, including postpartum psychosis. Other staff reflected on the contribution of the current viral pandemic to the content of delusions arising in the context of new-onset or pre-existing psychosis. Another theme was that of fears of COVID-19 infection: ‘Pregnant mothers extremely worried about the risk of catching COVID-19 and the risks to their babies’.

Other themes arose due to broader societal restrictions. Most staff reported concerns about loss of support and subsequent isolation in the context of reduced contact with family, friends and professionals during pregnancy and in the postpartum: ‘perinatal period … difficult time for some, involving some isolation. With the lockdown, many pregnant women were left having remote appointments’. Another closely related theme was concerns about those at an increased risk of domestic violence and abuse (DVA): ‘Vulnerable women and children at higher risk of DV’ and ‘Domestic abuse situations increasing’, alongside feelings from staff of powerlessness to intervene, which will be considered further in the section that follows.

Most participants reported increases in depression and anxiety disorders among perinatal women, including worsening or relapse of pre-pandemic disorders or new symptoms since the pandemic’s onset. One staff respondent wrote: ‘Anxiety and OCD [Obsessive Compulsive Disorder] symptoms related to fear of infection’. Another commented: ‘Impacting on mood and increasing risk of harm to self’, when reflecting on the impact of social isolation. A few staff noted an increase in substance use: ‘increased substance misuse among perinatal women’. Another theme which may have contributed to anxiety and depression in perinatal women was increased uncertainty and lack of control, already prominent during the perinatal period: ‘Feelings of being out of control’. Finally, a few staff reflected that women’s experiences of having a baby during the pandemic had been very different from what they had expected: ‘women due to give birth … very concerned about their birth experience’ and ‘Anxiety related to change in birth plans … Birth partner not being present at birth of baby’. A possible increased risk of birth trauma was noted by some staff: ‘Birth trauma due to restrictions in maternity services’*.* Many staff were particularly concerned about the post-traumatic disorder (PTSD) in perinatal women in the longer term.

### Changes to perinatal mental health care

Staff were also asked about changes made to services during the pandemic. 44.4% (92/207) of staff working in community teams estimated that referral rates declined by more than 10%. In inpatient services, 54.8% (17/31) of staff estimated that the monthly admission rate declined by more than 10%.

All respondents were asked to rate the relevance of challenges to current work in the pandemic (since mid-March 2020). These differed somewhat between inpatient and residential settings (hospital inpatient service, crisis houses and residential services) and community settings (CMHTs, crisis teams and community groups). Four challenges rated most frequently as very or extremely relevant in inpatient and residential settings were pressures of supporting colleagues (31/58; 53.4%), adapting to new ways of working (31/58; 53.4%), being infected with COVID-19 at work (30/58; 51.7%) and putting infection control measures into practice (28/58; 48.3%). The same challenges were identified in staff who reported working in MBUs specifically. In community settings, the most frequently rated as very or extremely relevant were adapting to new ways of working (188/307; 61.2%), learning to use new technologies too quickly and/or without sufficient training and support (147/306; 48%), responding to additional mental health needs arising from COVID-19 (138/308; 44.8%) and pressures of supporting colleagues (123/305; 40.3%).

As all staff in the sample reported working with the perinatal population, they were later presented with additional challenges specific to perinatal work. Table 3 displays the proportion of staff rating each item as ‘very relevant’ or ‘extremely relevant’; items are ordered by highest proportion first. Completion rates of these items were just under 60% of the sample.

**Table 3:** Challenges to perinatal work rated as very or extremely relevant by mental health staff

	*n*/N*	%
Challenges assessing mother and infant relationships because of lack of direct access	90/208	43.3
Difficulty planning and monitoring treatment due to reduced social care services	67/209	32.1
Difficulty planning and monitoring treatment due to reduced community midwife and health visitor services	62/211	29.4
Safeguarding procedures are more difficult than usual to mobilise	62/211	29.4
Reduced access to maternity units to carry out assessments	59/211	28.0
Referrals to our service not made or delayed because of the COVID-19 crisis	58/210	27.6
Reduced opportunities to admit to mother and baby units	37/206	18.0
Challenges arising from maternal or infant COVID-19 infection	35/207	16.9
Children are too readily taken into care because of obstacles to making other assessment and management plans at the present time	9/208	4.3

^*^Total respondents for this item

**Fig. 2:**
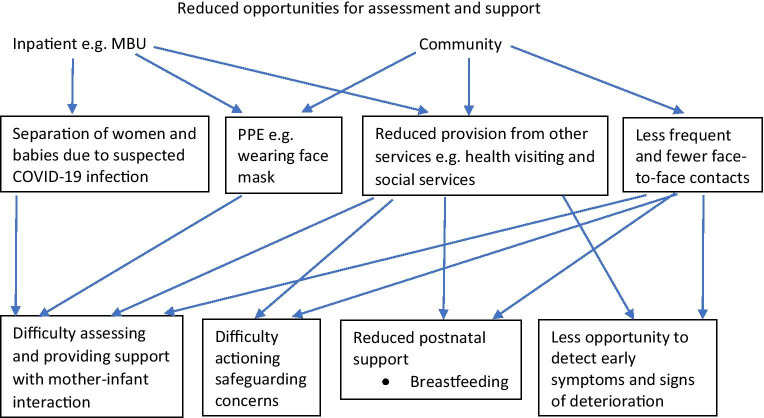
Challenges to perinatal mental health service provision expressed by staff (themes in boxes)

Some challenges related to reduced ability to adequately assess and support perinatal women, their babies and their wider families, were echoed in responses to open questions. Themes of changes staff were most concerned about in the perinatal setting and their perceived impact are displayed in Fig. 2. Community staff reported less frequent and fewer face-to-face contacts with women. Staff were concerned about their ability to detect early signs of mental illness when reviewing women remotely*.* Many were also worried about how to adequately assess and support the mother-infant interaction during the remote consultation by telephone or video: ‘unable to [do] vital work to help mums bond with their babies … only allowed to undertake phone calls not home visits.’ In extreme cases where staff are worried about the infant’s well-being and have safeguarding concerns, this assessment of interaction is part of the risk formulation as one staff respondent summarised: ‘Vulnerable babies being cared for by unwell mothers who are not being identified due to lockdown because they are more isolated and less likely to be picked up by professionals’. Risk assessment may also be challenging in a remote consultation: ‘Women with know[n] Domestic Abuse … doing video consultations so may not be able to be entirely honest about symptoms and risk’. Another barrier to actioning safeguarding concerns identified by some staff was reduced provision from other agencies such as social services. Another theme of reduced community service provision in the postnatal period from other services, such as health visiting, was identified, which some staff felt impacted on breastfeeding support: ‘not getting hands on support’.

Infection control measures presented some specific challenges in the inpatient perinatal setting to the assessment of the mother-infant interaction. A few staff working on MBUs reflected on the challenges posed by the separation of women and babies with suspected COVID-19. Others were concerned about the impact of face mask wearing on the developing infant: ‘babies … cared for by masked nurses … lack of interaction with facial features.’

Other open-ended questions elicited reflections on positive aspects of adaptations made to manage the impact which some staff felt would be beneficial to continue beyond the pandemic. Two themes of telemedicine benefits to service users and staff were identified. Several staff reflected on the flexibility of virtual appointments for women as busy new mothers and the range of online self-help and other supports available, such as cognitive behavioural therapy, mindfulness and third sector initiatives: ‘local and national groups such as PANDAS stepping up with online support.’ Benefits to staff were related to time management; reduced travel when working from home resulted in increased time for clinical record keeping and improved work-life balance.

## Discussion

### Main findings

Mental health staff in this survey, administered during the COVID-19 pandemic lockdown in the UK, reported particular concerns about the mental health of women in the perinatal period. Staff who reported working with perinatal women more often endorsed as relevant for service users those items pertaining to social isolation and abusive relationships than in the full sample of mental health staff surveyed (Johnson et al. 2020). While there has been little published research on the impact of the pandemic on perinatal mental health, pregnant women in China (Liu et al. 2020; Wu et al. 2020) and Canada (Saccone et al. 2020) assessed following, versus those assessed prior to, the onset of the pandemic, reported greater levels of anxiety and depression symptoms. However, to our knowledge, there has been no study to date reporting on staff perceptions of mental health care for perinatal women.

Staff working in the perinatal setting were concerned about how to support women during pregnancy and in the postpartum period as a result of changes to care provided by their own and other services. Changes included reduced face-to-face contact in community mental health services, changes in infection control measures and reduced social care and home visiting services (i.e. maternity and health visiting services). Consequently, staff were worried about how to mobilise safeguarding procedures and assess and support the mother-infant interaction. There were also concerns about the impact of wearing masks on the developing infant; to our knowledge, there has been no research investigating the impact of this in residential settings such as MBUs. However, staff highlighted positive changes such as the greater flexibility provided by remote consulting.

The impact of COVID-19 infection on women and their infants was inevitably a concern. Neuropsychiatric complications of infection have been reported, including acute confusion (Rogers et al. 2020), which may mimic postpartum psychosis.

### Strengths and limitations

This is the first study to document the experiences of mental health care staff supporting women in the perinatal period during the COVID-19 pandemic. The use of both qualitative and quantitative data facilitates the triangulation of our findings (Tracy 2010).

However, a limitation of surveys is that they may overrepresent those who are particularly distressed and keen to voice their concerns. Another limitation is that it was not possible to verify that respondents were definitely mental health professionals. There are also some limitations posed by the convenient nature of the sample. Despite efforts to recruit a broad ethnic demographic, the number of non-White respondents in this sample is relatively small. The sample is mainly female and there may be gendered attitudes which are overrepresented. There is also limited geographical diversity, with most of the respondents being from England; there may be different experiences in the UK’s devolved nations, with differing levels of perinatal mental health service provision, which were not adequately captured. Most of the staff were working in community services, with only 18 respondents from the MBU. There were also no questions about the experiences of partners of perinatal women so these experiences were not captured.

Furthermore, the sample included both staff working only in perinatal services and other staff working with perinatal women among other service users or in non-perinatal settings. Yet, we believe the latter group is more representative of the structure of health services in many places outside the UK where there are no dedicated perinatal mental health services.

### Implications

As has been expressed by others (Holmes et al. 2020; Johnson et al. 2020) and highlighted in this study, some changes to service delivery will suit some groups of service users more than others so support must continue to be individually tailored. Indeed our study highlighted challenges to remote consulting unique to the perinatal period. Face-to-face assessment is necessary in high-risk cases as highlighted by the recent confidential enquiry of maternal deaths in the UK during the first 3 months of the pandemic, which included four suicides and two domestic homicides (Knight et al. 2020).

However, the findings of this study have also highlighted some of the advantages of perinatal mental health care using virtual platforms. During and beyond the current pandemic, there is potential for the flexibility provided by remote consulting to reduce non-attendance rates (Rose et al. 2020). Despite this, interviews with women in maternity settings in the UK during the pandemic suggest that the use of virtual appointments made them reluctant to discuss mental health issues (Karavadra et al. 2020).

Future research would usefully focus on how to manage these particular areas of difficulty such as the assessment of perinatal mental health, DVA and the mother-infant relationship to equip staff with the tools to support women remotely and increase awareness of the circumstances under which remote assessment may be inappropriate. Further research to elucidate the direct effect of COVID-19 infection on pregnant and breastfeeding women and the developing infant (Juan et al. 2020) would also help to address another major stressor for women during the pandemic. This would also allow perinatal mental health staff to provide women with accurate information about the risks posed by COVID-19 to reduce the uncertainty contributing to increased anxiety among the perinatal population during this time (Motrico et al. 2020).

### Conclusions

In summary, there is an urgent need for research that provides an understanding of the experiences of perinatal women and their families during the COVID-19 pandemic, including potential barriers to supporting families during this time. Greater understanding could illuminate potential targets for interventions by mental health staff and other professionals to support women with their perinatal mental health and safeguard women and their families against violence and abuse during the current pandemic and beyond.

**Table:** 

Lived experience commentary: Clare Dolman and Sarah Spring.
This survey highlights the challenges encountered by mental health professionals trying to deliver vital services during the pandemic but reading their views on what they *thought* had affected women with perinatal mental illness the most during the pandemic, we wondered what women *themselves* would say had been most relevant to them. I understand that another survey is being conducted to answer those very questions, but perhaps that’s worth pointing out here.
There was no specific mention of the impact on fathers which one might expect when dealing with a perinatal population, though a couple of ‘difficulties’ related to families/carers. The results illustrate the detrimental effect of lack of face-to-face consultations, especially important to help women having problems with breastfeeding and bonding with their baby. Using video calls is referred to as a possible solution but they rely on a good connection, availability of the technology and a woman’s ability to use such technology if cognitively challenged when struggling with depression or other mental illness so reliance on this medium may exclude many mothers. For those women with developing psychosis and other conditions involving hallucinations, contact other than face-to-face could exacerbate or precipitate more delusions and, as mothers with these illnesses are good at disguising their symptoms, the severity of their illness could go undetected. This is another reason why remote contact should be used with extreme caution.
In relation to the service difficulties identified during the pandemic, it is noteworthy that perinatal services weren’t designated an essential service. This is an important point which should be considered in any future lockdown: many perinatal team staff were redeployed to hospital roles so women who had been considered serious enough to receive weekly therapeutic visits were only seen much less frequently thus increasing the risk that their condition would worsen. Similarly numbers of Health Visitors, who provide essential support to women in the perinatal period, were redeployed. This policy now appears short-sighted and in need of review.
This is an independently written perspective from lived experience contributed by some of the members of the IOPPN’s Women’s Mental Health Section’s service user perinatal advisory group with relevant experience.

## Data Availability

The survey dataset is currently being used for additional research by the author research group and is not currently available in a data repository. A copy of the survey is available at this web address: https://opinio.ucl.ac.uk/s?s=67819.

## Declarations

### Disclaimer

The views expressed are those of the authors and not necessarily those of the NIHR, the Department of Health and Social Care or its arm's length bodies or other government departments.
